# Crystal structure of 5,5′-[(4-fluoro­phen­yl)methyl­ene]bis­[6-amino-1,3-di­methyl­pyrimidine-2,4(1*H*,3*H*)-dione]

**DOI:** 10.1107/S1600536814019886

**Published:** 2014-09-10

**Authors:** Naresh Sharma, Goutam Brahmachari, Bubun Banerjee, Rajni Kant, Vivek K. Gupta

**Affiliations:** a Post-Graduate Department of Physics & Electronics, University of Jammu, Jammu Tawi 180 006, India; b Laboratory of Natural Products & Organic Synthesis, Department of Chemistry, Visva-Bharati University, Santiniketan 731 235, West Bengal, India

**Keywords:** crystal structure, uracil derivatives, biological activity, pyrimidine scaffolds, bis-uracil derivatives

## Abstract

In the title mol­ecule, C_19_H_21_FN_6_O_4_, the dihedral angles between the benzene ring and essentially planar pyrimidine rings [maximum deviations of 0.036 (2) and 0.056 (2) Å] are 73.32 (7) and 63.81 (8)°. The dihedral angle between the mean planes of the pyrimidine rings is 61.43 (6)°. In the crystal, N—H⋯O hydrogen bonds link mol­ecules, forming a two-dimensional network parallel to (001) and in combination with weak C—H⋯O hydrogen bonds, a three-dimensional network is formed. Weak C—H⋯π inter­actions and π–π inter­actions, with a centroid–centroid distance of 3.599 (2) Å are also observed.

## Related literature   

For the biological activity of uracil derivatives, see: Muller *et al.* (1993 ▶); Buckle *et al.* (1994 ▶). For drugs containing purine moieties, see: Zhi *et al.* (2003 ▶); Devi & Bhuyan (2005 ▶). For the biological activity of pyrimidine scaffolds, see: Makarov *et al.* (2005 ▶); Deshmukh *et al.* (2009 ▶); Ibrahim & El-Metwally (2010 ▶). For the synthesis of bis-uracil derivatives, see: Karimi *et al.* (2013 ▶). For a related structure, see: Das *et al.* (2009 ▶).
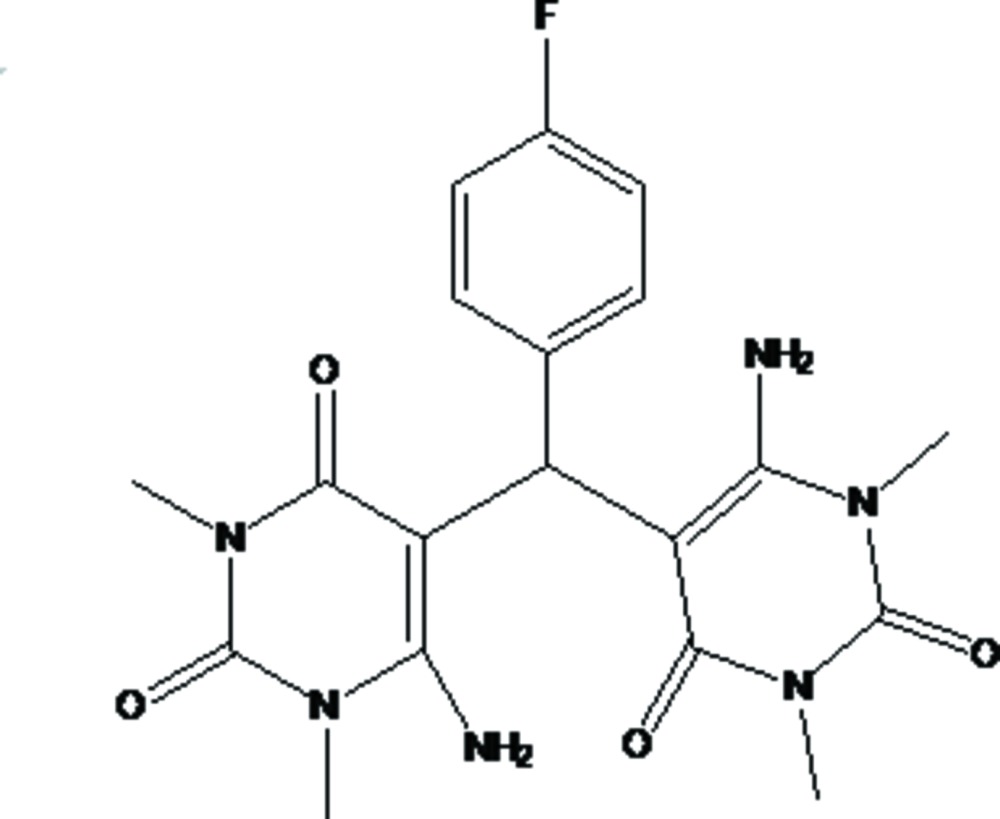


## Experimental   

### Crystal data  

C_19_H_21_FN_6_O_4_
*M* *_r_* = 416.42
Orthorhombic, 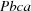

*a* = 14.6208 (6) Å
*b* = 11.3324 (7) Å
*c* = 22.6410 (12) Å
*V* = 3751.4 (3) Å^3^
*Z* = 8
Mo *K*α radiation
μ = 0.11 mm^−1^
*T* = 293 K
0.30 × 0.20 × 0.20 mm

### Data collection  

Oxford Diffraction Xcalibur Sapphire3 diffractometer
Absorption correction: multi-scan (*CrysAlis RED*; Oxford Diffraction, 2010 ▶) *T*_min_ = 0.862, *T*_max_ = 1.000
9655 measured reflections
3665 independent reflections
2208 reflections with *I* > 2σ(*I*)
*R*_int_ = 0.047

### Refinement  

*R*[*F* ^2^ > 2σ(*F* ^2^)] = 0.052
*wR*(*F* ^2^) = 0.130
*S* = 1.04
3665 reflections
291 parameters
H atoms treated by a mixture of independent and constrained refinement
Δρ_max_ = 0.19 e Å^−3^
Δρ_min_ = −0.20 e Å^−3^

### 

Data collection: *CrysAlis PRO* (Oxford Diffraction, 2010 ▶); cell refinement: *CrysAlis PRO*; data reduction: *CrysAlis PRO*; program(s) used to solve structure: *SHELXS97* (Sheldrick, 2008 ▶); program(s) used to refine structure: *SHELXL97* (Sheldrick, 2008 ▶); molecular graphics: *ORTEP-3 for Windows* (Farrugia, 2012 ▶); software used to prepare material for publication: *PLATON* (Spek, 2009 ▶).

## Supplementary Material

Crystal structure: contains datablock(s) I, New_Global_Publ_Block. DOI: 10.1107/S1600536814019886/lh5725sup1.cif

Structure factors: contains datablock(s) I. DOI: 10.1107/S1600536814019886/lh5725Isup2.hkl

Supporting information file. DOI: 10.1107/S1600536814019886/lh5725Isup3.cml

**Fig. 1.**
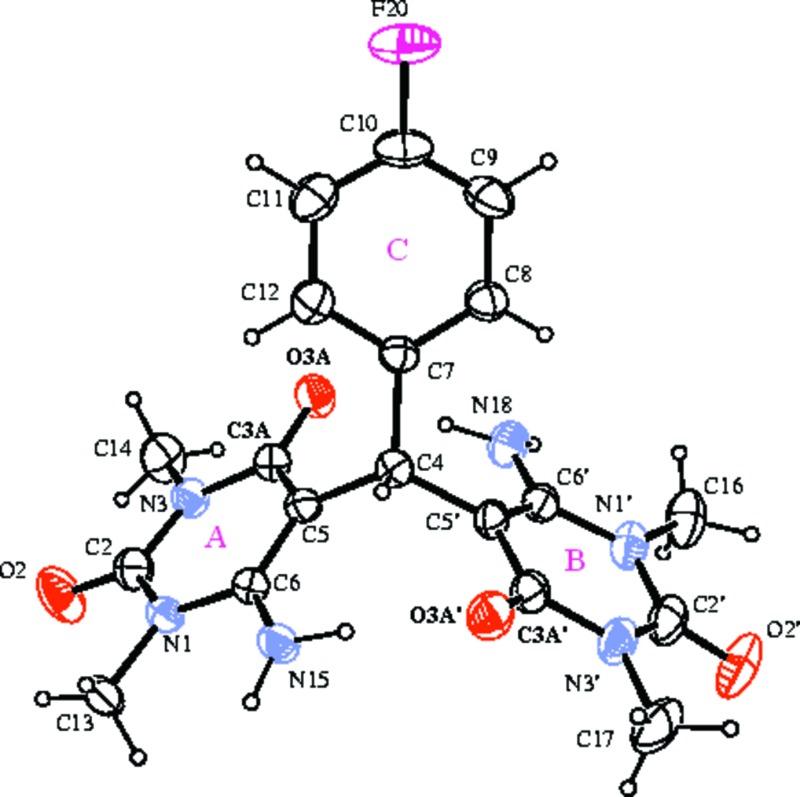
The molecular structure with displacement ellipsoids drawn at the 40% probability level. H atoms are shown as small spheres of arbitrary radii.

**Fig. 2.**
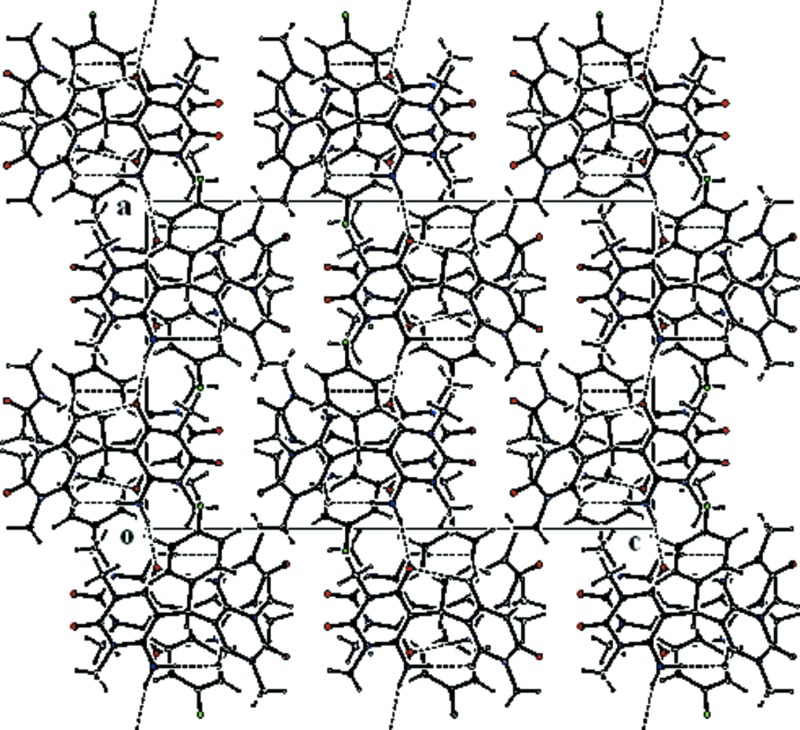
Part of the crystal structure viewed along the a axis. Hydrogen bonds are shown as dashed lines.

. DOI: 10.1107/S1600536814019886/lh5725fig1.tif
The mol­ecular structure with displacement ellipsoids drawn at the 40% probability level. H atoms are shown as small spheres of arbitrary radii.

a . DOI: 10.1107/S1600536814019886/lh5725fig2.tif
Part of the crystal structure viewed along the *a* axis. Hydrogen bonds are shown as dashed lines.

CCDC reference: 973485

Additional supporting information:  crystallographic information; 3D view; checkCIF report

## Figures and Tables

**Table 1:** Hydrogen-bond geometry (Å, °) *Cg* is the centroid of the C7–C12 ring.

*D*—H⋯*A*	*D*—H	H⋯*A*	*D*⋯*A*	*D*—H⋯*A*
N15—H40⋯O3*A*′	0.96 (3)	1.96 (3)	2.916 (3)	174 (2)
N18—H50⋯O3*A*	0.93 (3)	1.88 (3)	2.803 (3)	170 (2)
N15—H30⋯O3*A* ^i^	0.86 (3)	2.26 (3)	3.083 (3)	161 (3)
N18—H60⋯O3*A*′^ii^	0.91 (3)	2.14 (3)	3.007 (3)	159 (2)
C13—H13*A*⋯O3*A* ^i^	0.96	2.41	3.154 (3)	134
C13—H13*A*⋯*Cg* ^iii^	0.96	2.98	3.744 (3)	138

Symmetry codes: (i) 
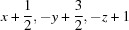
; (ii) 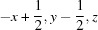; (iii) 
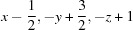
.

## supplementary crystallographic information

### S2. Structural commentary

Uracil derivatives represent a "privileged" structural motif in a wide variety of natural and synthetic compounds with a broad spectrum of significant biological activities (Muller *et al.*, 1993). 6-Amino­uracils are the important starting compounds for the synthesis of medicinally useful xanthines and theophyllines, which are now routinely used as a phospho­diesterase inhibitor for the treatment of asthma (Buckle *et al.*, 1994). 6-Amino­uracils are regarded as the key inter­mediates for the synthesis of purine-based drugs, such as penciclovir, caffeine, theophylline, and theobromine (Zhi *et al.*, (2003); Devi & Bhuyan, 2005). In addition, pyrimidine scaffolds are reported to exhibit diverse biological and pharmaceutical activities (Ibrahim & El-Metwally, 2010; Deshmukh *et al.*, 2009), Makarov *et al.*, 2005). Herein, we report the synthesis and crystal structure of a new aryl­methyl­ene-bis uracil derivative, namely 5,5'-((4-fluoro­phenyl)­methyl­ene) bis­(6-amino-1,3-di­methyl­pyrimidine-2,4(1*H*,3H)-dione) synthesized *via* one-pot pseudo multicomponent reaction at room temperature using iodine as inexpensive and eco-friendly catalyst.

The molecular structure of the title compound is shown in Fig. 1. The distances are in the normal ranges and correspond to those observed in a related structure (Das *et al.*, 2009). The pyrimidine rings are essentially planar with maximum deviations of 0.036 (2) and 0.056 (2) Å for C6 and N1', respectively. The dihedral angle between the mean plane of benzene ring [C7—C12] and pyrimidine rings-A and B are 73.32 (7) ° and 63.81 (8) ° respectively. The dihedral angle between the two pyrimidine rings is 61.43 (6) °. The planarity of the phenyl group confirms its aromatic character. From the least-squares plane calculations of the phenyl moiety, the maximum deviation observed is 0.014 (2) Å for atom C8. The double bond distances C2—O2 = 1.220 (4) Å, C3—O3A = 1.257 (3) Å (ring-A) and C2'- O2' = 1.217 (4) Å, C3A'- O3A' = 1.261 (4) Å (ring-B), are significantly larger than the standard value for carbonyl group (1.192 Å) and lengthening of the C═O double bond is due their involvment in N—H···O and C—H···O hydrogen bonds. In the crystal, N—H···O hydrogen bonds link molecules forming a two-dimensional network parallel to (001) (Fig. 2) and in combination with weak C—H···O hydrogen bonds a three-dimensional network is formed. Weak C—H···π inter­actions and π–π inter­actions with a centroid–centroid distance of 3.599 (2) Å between pyrimidine ring-B and benzene ring-C at (1/2 - *x*, -1/2 + *y*, *z*) are also observed.

### S5. Synthesis and crystallization

An oven-dried screw cap test tube was charged with a magnetic stir bar, 6-amino-1,3-di­methyl­uracil (0.155 g, 1.0 mmol), 4-fluoro­benzaldehyde (0.062 g, 0.5 mmol), iodine (0.025 g, 10 mol % as catalyst), and EtOH:H_2_O (1:1 *v*/*v*; 4 ml) in a sequential manner. The reaction mixture was then stirred vigorously at room temperature and the stirring was continued for 4 h; the progress of the reaction was monitored by TLC. On completion of the reaction, a solid mass precipitated out, which was filtered, and washed with aqueous ethanol to obtain the crude product that was purified just by recrystallization from ethanol without carrying out column chromatography (72% yield). The title compound forms as a White solid. Yield 72%. Mp: 537–539 K. IR (KBr) ν_max_ cm^-1^: 3430, 3104, 2956, 1690, 1603, 1498, 1247, 1216, 1142, 1070, 870, 789, 756. ^1^H NMR (400 MHz, DMSO-d_6_) δ/p.p.m.: 7.42 (4*H*, s, –NH_2_), 7.11 (2*H*, dd, *J* = 8.4 & 5.6 Hz, aromatic H), 6.99 (2*H*, t, *J* = 8.8 Hz, aromatic H), 5.56 (1*H*, s, –CH–), 3.32 (6*H*, s, 2 × NCH_3_), 3.14 (6*H*, s, 2 × NCH_3_). TOF-MS: 439.1513 [*M*+Na]^+^. The structure of 5,5'-((4-fluoro­phenyl)­methyl­ene)bis­(6-amino-1, 3-di­methyl­pyrimidine-2,4(1*H*,3H)-dione) was characterized by means of spectral studies including FT—IR, ^1^H NMR, and TOF-MS. Crystals suitable for X-ray diffraction were grown by dissolving 50 mg of the title compound in 5 ml DMSO and after several days at ambient temperature colourless block-shaped crystals were formed.

### S6. Refinement

Crystal data, data collection and structure refinement details are summarized in Table 1. Atoms H30, H40 attached to N15 and H50, H60 attached to N18 were located in a difference Fourier map and refined isotropically. All the remaining H atoms were geometrically fixed and allowed to ride on their parent C atoms, with C—H distances of 0.93–0.98 Å; and with *U*_iso_(H) = 1.2*U*_eq_(C), except for the methyl group where *U*_iso_(H) = 1.5*U*_eq_(C).

### Crystal data

**Table:**

C_19_H_21_FN_6_O_4_	*F*(000) = 1744
*M**_r_* = 416.42	*D*_x_ = 1.475 Mg m^−^^3^
Orthorhombic, *P**b**c**a*	Mo *K*α radiation, λ = 0.71073 Å
Hall symbol: -P 2ac 2ab	Cell parameters from 2766 reflections
*a* = 14.6208 (6) Å	θ = 4.0–29.0°
*b* = 11.3324 (7) Å	µ = 0.11 mm^−^^1^
*c* = 22.6410 (12) Å	*T* = 293 K
*V* = 3751.4 (3) Å^3^	Rectangular, white
*Z* = 8	0.30 × 0.20 × 0.20 mm

### Data collection

**Table:**

Oxford Diffraction Xcalibur Sapphire3 diffractometer	3665 independent reflections
Radiation source: fine-focus sealed tube	2208 reflections with *I* > 2σ(*I*)
Graphite monochromator	*R*_int_ = 0.047
Detector resolution: 16.1049 pixels mm^-1^	θ_max_ = 26.0°, θ_min_ = 3.4°
ω scans	*h* = −17→18
Absorption correction: multi-scan (*CrysAlis RED*; Oxford Diffraction, 2010)	*k* = −13→10
*T*_min_ = 0.862, *T*_max_ = 1.000	*l* = −27→27
9655 measured reflections	

### Refinement

**Table:**

Refinement on *F*^2^	Primary atom site location: structure-invariant direct methods
Least-squares matrix: full	Secondary atom site location: difference Fourier map
*R*[*F*^2^ > 2σ(*F*^2^)] = 0.052	Hydrogen site location: inferred from neighbouring sites
*wR*(*F*^2^) = 0.130	H atoms treated by a mixture of independent and constrained refinement
*S* = 1.04	*w* = 1/[σ^2^(*F*_o_^2^) + (0.0402*P*)^2^ + 0.280*P*] where *P* = (*F*_o_^2^ + 2*F*_c_^2^)/3
3665 reflections	(Δ/σ)_max_ < 0.001
291 parameters	Δρ_max_ = 0.19 e Å^−^^3^
0 restraints	Δρ_min_ = −0.20 e Å^−^^3^

### Special details

**Table:**

Experimental. *CrysAlis PRO*, Agilent Technologies, Version 1.171.36.28 (release 01–02-2013 CrysAlis171. NET) (compiled Feb 1 2013,16:14:44) Empirical absorption correction using spherical harmonics, implemented in SCALE3 ABSPACK scaling algorithm.
Geometry. All e.s.d.'s (except the e.s.d. in the dihedral angle between two l.s. planes) are estimated using the full covariance matrix. The cell e.s.d.'s are taken into account individually in the estimation of e.s.d.'s in distances, angles and torsion angles; correlations between e.s.d.'s in cell parameters are only used when they are defined by crystal symmetry. An approximate (isotropic) treatment of cell e.s.d.'s is used for estimating e.s.d.'s involving l.s. planes.
Refinement. Refinement of *F*^2^ against ALL reflections. The weighted *R*-factor *wR* and goodness of fit *S* are based on *F*^2^, conventional *R*-factors *R* are based on *F*, with *F* set to zero for negative *F*^2^. The threshold expression of *F*^2^ > σ(*F*^2^) is used only for calculating *R*-factors(gt) *etc*. and is not relevant to the choice of reflections for refinement. *R*-factors based on *F*^2^ are statistically about twice as large as those based on *F*, and *R*- factors based on ALL data will be even larger.

### Fractional atomic coordinates and isotropic or equivalent isotropic displacement parameters (Å^2^)

**Table:**

	*x*	*y*	*z*	*U*_iso_*/*U*_eq_	
O3A	0.12040 (11)	0.70286 (16)	0.47999 (8)	0.0448 (5)	
O3A'	0.41972 (12)	0.90382 (17)	0.35851 (8)	0.0481 (5)	
N3	0.21150 (13)	0.67789 (19)	0.56011 (9)	0.0385 (5)	
N1'	0.27279 (15)	0.63863 (19)	0.28860 (9)	0.0445 (6)	
C5'	0.28708 (16)	0.7846 (2)	0.36460 (10)	0.0336 (6)	
N1	0.35782 (12)	0.7609 (2)	0.56448 (9)	0.0388 (5)	
C5	0.26609 (15)	0.7945 (2)	0.47801 (10)	0.0317 (6)	
N18	0.16272 (16)	0.6468 (2)	0.36253 (12)	0.0445 (6)	
O2	0.29971 (14)	0.6586 (2)	0.64250 (9)	0.0697 (7)	
C4	0.25464 (16)	0.8542 (2)	0.41822 (10)	0.0337 (6)	
H4	0.2992	0.9187	0.4203	0.040*	
C6	0.34765 (15)	0.8050 (2)	0.50801 (10)	0.0328 (6)	
C6'	0.24099 (18)	0.6903 (2)	0.34020 (11)	0.0374 (6)	
N15	0.42224 (15)	0.8593 (2)	0.48533 (11)	0.0410 (6)	
C7	0.16385 (16)	0.9204 (2)	0.41091 (11)	0.0323 (6)	
C3A'	0.37186 (18)	0.8189 (2)	0.34004 (11)	0.0393 (6)	
N3'	0.40473 (15)	0.7555 (2)	0.29207 (9)	0.0465 (6)	
C3A	0.19584 (17)	0.7257 (2)	0.50409 (11)	0.0366 (6)	
F20	−0.06866 (11)	1.12341 (16)	0.39325 (9)	0.0834 (7)	
C2	0.28952 (18)	0.6969 (3)	0.59256 (12)	0.0437 (7)	
O2'	0.39032 (16)	0.60699 (19)	0.22495 (9)	0.0746 (7)	
C2'	0.3585 (2)	0.6640 (3)	0.26558 (12)	0.0503 (8)	
C13	0.44207 (17)	0.7825 (3)	0.59804 (11)	0.0499 (8)	
H13A	0.4918	0.7404	0.5801	0.075*	
H13B	0.4341	0.7558	0.6380	0.075*	
H13C	0.4554	0.8655	0.5980	0.075*	
C12	0.13307 (17)	0.9921 (2)	0.45682 (12)	0.0441 (7)	
H12	0.1651	0.9932	0.4923	0.053*	
C8	0.11561 (17)	0.9230 (2)	0.35843 (12)	0.0405 (7)	
H8	0.1362	0.8786	0.3265	0.049*	
C10	0.00938 (18)	1.0575 (2)	0.39853 (15)	0.0505 (8)	
C11	0.05596 (19)	1.0616 (3)	0.45075 (14)	0.0510 (8)	
H11	0.0363	1.1098	0.4815	0.061*	
C9	0.03704 (18)	0.9907 (2)	0.35245 (14)	0.0502 (8)	
H9	0.0040	0.9900	0.3174	0.060*	
C14	0.14123 (18)	0.6033 (3)	0.58682 (13)	0.0536 (8)	
H14A	0.1677	0.5579	0.6183	0.080*	
H14B	0.1167	0.5509	0.5575	0.080*	
H14C	0.0931	0.6519	0.6022	0.080*	
C17	0.4962 (2)	0.7837 (3)	0.26916 (14)	0.0683 (10)	
H17A	0.5409	0.7351	0.2885	0.102*	
H17B	0.5096	0.8653	0.2766	0.102*	
H17C	0.4979	0.7690	0.2274	0.102*	
C16	0.2188 (2)	0.5502 (3)	0.25638 (13)	0.0626 (9)	
H16A	0.2378	0.5486	0.2158	0.094*	
H16B	0.1551	0.5704	0.2585	0.094*	
H16C	0.2283	0.4739	0.2738	0.094*	
H60	0.1393 (18)	0.576 (3)	0.3509 (12)	0.057 (9)*	
H50	0.1483 (16)	0.675 (2)	0.4000 (13)	0.049 (8)*	
H40	0.4206 (17)	0.880 (2)	0.4441 (13)	0.060 (9)*	
H30	0.476 (2)	0.856 (3)	0.5011 (13)	0.069 (10)*	

### Atomic displacement parameters (Å^2^)

**Table:**

	*U*^11^	*U*^22^	*U*^33^	*U*^12^	*U*^13^	*U*^23^
O3A	0.0359 (10)	0.0609 (13)	0.0376 (10)	−0.0095 (9)	−0.0013 (8)	0.0047 (10)
O3A'	0.0439 (10)	0.0557 (12)	0.0446 (11)	−0.0059 (10)	0.0066 (9)	0.0039 (11)
N3	0.0360 (11)	0.0470 (14)	0.0326 (11)	−0.0023 (10)	0.0028 (10)	0.0068 (12)
N1'	0.0635 (15)	0.0365 (13)	0.0335 (12)	0.0040 (11)	0.0064 (11)	0.0008 (11)
C5'	0.0385 (14)	0.0364 (15)	0.0259 (12)	0.0042 (12)	0.0025 (11)	0.0036 (12)
N1	0.0311 (11)	0.0530 (15)	0.0322 (11)	0.0047 (10)	−0.0026 (9)	0.0040 (12)
C5	0.0263 (12)	0.0375 (15)	0.0313 (12)	0.0021 (11)	0.0015 (11)	0.0023 (12)
N18	0.0511 (15)	0.0398 (15)	0.0424 (14)	−0.0043 (12)	0.0052 (13)	−0.0045 (13)
O2	0.0658 (14)	0.1013 (19)	0.0420 (12)	−0.0168 (13)	−0.0105 (11)	0.0299 (13)
C4	0.0340 (13)	0.0350 (15)	0.0320 (13)	0.0015 (12)	−0.0005 (11)	0.0012 (12)
C6	0.0320 (13)	0.0355 (14)	0.0310 (13)	0.0041 (11)	−0.0008 (11)	−0.0001 (12)
C6'	0.0450 (15)	0.0349 (15)	0.0324 (13)	0.0090 (13)	0.0004 (12)	0.0048 (13)
N15	0.0305 (12)	0.0565 (15)	0.0361 (13)	−0.0035 (11)	−0.0025 (11)	0.0055 (12)
C7	0.0302 (13)	0.0315 (14)	0.0352 (14)	0.0001 (11)	0.0000 (11)	0.0043 (12)
C3A'	0.0466 (16)	0.0409 (16)	0.0306 (13)	0.0059 (13)	0.0038 (13)	0.0092 (14)
N3'	0.0534 (14)	0.0510 (15)	0.0349 (12)	0.0043 (12)	0.0139 (11)	0.0073 (12)
C3A	0.0380 (14)	0.0399 (16)	0.0320 (13)	0.0041 (12)	0.0000 (12)	−0.0005 (13)
F20	0.0666 (12)	0.0682 (13)	0.1154 (17)	0.0373 (10)	−0.0044 (11)	0.0026 (13)
C2	0.0443 (16)	0.0500 (18)	0.0366 (15)	0.0012 (14)	0.0019 (13)	0.0050 (15)
O2'	0.1086 (18)	0.0632 (15)	0.0521 (13)	0.0067 (13)	0.0384 (13)	−0.0086 (13)
C2'	0.071 (2)	0.0446 (18)	0.0354 (15)	0.0101 (16)	0.0139 (15)	0.0097 (15)
C13	0.0360 (14)	0.078 (2)	0.0360 (14)	0.0019 (15)	−0.0102 (12)	0.0074 (16)
C12	0.0468 (16)	0.0436 (17)	0.0420 (16)	0.0021 (13)	0.0006 (13)	−0.0035 (14)
C8	0.0459 (15)	0.0344 (15)	0.0414 (15)	0.0067 (12)	−0.0041 (13)	0.0006 (13)
C10	0.0418 (16)	0.0333 (16)	0.076 (2)	0.0096 (13)	0.0004 (16)	0.0073 (17)
C11	0.0578 (18)	0.0398 (17)	0.0554 (19)	0.0045 (14)	0.0075 (16)	−0.0067 (16)
C9	0.0519 (17)	0.0431 (17)	0.0556 (18)	0.0095 (14)	−0.0143 (15)	0.0051 (16)
C14	0.0539 (18)	0.060 (2)	0.0474 (17)	−0.0143 (15)	0.0038 (14)	0.0125 (17)
C17	0.0648 (19)	0.082 (3)	0.0577 (18)	0.0027 (18)	0.0337 (16)	0.008 (2)
C16	0.095 (3)	0.0499 (19)	0.0431 (17)	−0.0025 (18)	0.0072 (17)	−0.0099 (17)

### Geometric parameters (Å, º)

**Table:**

O3A—C3A	1.257 (3)	C7—C12	1.394 (3)
O3A'—C3A'	1.261 (3)	C3A'—N3'	1.388 (3)
N3—C2	1.374 (3)	N3'—C2'	1.376 (3)
N3—C3A	1.398 (3)	N3'—C17	1.470 (3)
N3—C14	1.462 (3)	F20—C10	1.369 (3)
N1'—C2'	1.387 (3)	O2'—C2'	1.217 (3)
N1'—C6'	1.387 (3)	C13—H13A	0.9600
N1'—C16	1.469 (3)	C13—H13B	0.9600
C5'—C6'	1.379 (3)	C13—H13C	0.9600
C5'—C3A'	1.413 (3)	C12—C11	1.382 (4)
C5'—C4	1.523 (3)	C12—H12	0.9300
N1—C6	1.381 (3)	C8—C9	1.388 (3)
N1—C2	1.388 (3)	C8—H8	0.9300
N1—C13	1.468 (3)	C10—C9	1.351 (4)
C5—C6	1.377 (3)	C10—C11	1.365 (4)
C5—C3A	1.419 (3)	C11—H11	0.9300
C5—C4	1.522 (3)	C9—H9	0.9300
N18—C6'	1.345 (3)	C14—H14A	0.9600
N18—H60	0.91 (3)	C14—H14B	0.9600
N18—H50	0.93 (3)	C14—H14C	0.9600
O2—C2	1.220 (3)	C17—H17A	0.9600
C4—C7	1.533 (3)	C17—H17B	0.9600
C4—H4	0.9800	C17—H17C	0.9600
C6—N15	1.354 (3)	C16—H16A	0.9600
N15—H40	0.96 (3)	C16—H16B	0.9600
N15—H30	0.87 (3)	C16—H16C	0.9600
C7—C8	1.382 (3)		
			
C2—N3—C3A	124.1 (2)	O2—C2—N3	122.8 (3)
C2—N3—C14	116.9 (2)	O2—C2—N1	121.5 (3)
C3A—N3—C14	119.0 (2)	N3—C2—N1	115.7 (2)
C2'—N1'—C6'	122.1 (2)	O2'—C2'—N3'	122.8 (3)
C2'—N1'—C16	116.1 (2)	O2'—C2'—N1'	121.3 (3)
C6'—N1'—C16	121.8 (2)	N3'—C2'—N1'	115.9 (2)
C6'—C5'—C3A'	119.0 (2)	N1—C13—H13A	109.5
C6'—C5'—C4	124.6 (2)	N1—C13—H13B	109.5
C3A'—C5'—C4	116.4 (2)	H13A—C13—H13B	109.5
C6—N1—C2	122.4 (2)	N1—C13—H13C	109.5
C6—N1—C13	120.6 (2)	H13A—C13—H13C	109.5
C2—N1—C13	117.0 (2)	H13B—C13—H13C	109.5
C6—C5—C3A	118.0 (2)	C11—C12—C7	121.4 (3)
C6—C5—C4	119.7 (2)	C11—C12—H12	119.3
C3A—C5—C4	122.3 (2)	C7—C12—H12	119.3
C6'—N18—H60	122.2 (17)	C7—C8—C9	121.2 (3)
C6'—N18—H50	114.3 (16)	C7—C8—H8	119.4
H60—N18—H50	119 (2)	C9—C8—H8	119.4
C5—C4—C5'	116.4 (2)	C9—C10—C11	122.6 (3)
C5—C4—C7	114.11 (19)	C9—C10—F20	119.2 (3)
C5'—C4—C7	115.9 (2)	C11—C10—F20	118.2 (3)
C5—C4—H4	102.5	C10—C11—C12	118.3 (3)
C5'—C4—H4	102.5	C10—C11—H11	120.9
C7—C4—H4	102.5	C12—C11—H11	120.9
N15—C6—C5	123.3 (2)	C10—C9—C8	118.8 (3)
N15—C6—N1	115.4 (2)	C10—C9—H9	120.6
C5—C6—N1	121.2 (2)	C8—C9—H9	120.6
N18—C6'—C5'	123.3 (2)	N3—C14—H14A	109.5
N18—C6'—N1'	116.6 (2)	N3—C14—H14B	109.5
C5'—C6'—N1'	120.1 (2)	H14A—C14—H14B	109.5
C6—N15—H40	117.4 (16)	N3—C14—H14C	109.5
C6—N15—H30	124 (2)	H14A—C14—H14C	109.5
H40—N15—H30	116 (3)	H14B—C14—H14C	109.5
C8—C7—C12	117.6 (2)	N3'—C17—H17A	109.5
C8—C7—C4	123.0 (2)	N3'—C17—H17B	109.5
C12—C7—C4	119.0 (2)	H17A—C17—H17B	109.5
O3A'—C3A'—N3'	117.6 (2)	N3'—C17—H17C	109.5
O3A'—C3A'—C5'	124.5 (2)	H17A—C17—H17C	109.5
N3'—C3A'—C5'	118.0 (2)	H17B—C17—H17C	109.5
C2'—N3'—C3A'	124.1 (2)	N1'—C16—H16A	109.5
C2'—N3'—C17	117.2 (2)	N1'—C16—H16B	109.5
C3A'—N3'—C17	118.6 (2)	H16A—C16—H16B	109.5
O3A—C3A—N3	117.2 (2)	N1'—C16—H16C	109.5
O3A—C3A—C5	124.6 (2)	H16A—C16—H16C	109.5
N3—C3A—C5	118.2 (2)	H16B—C16—H16C	109.5
			
C6—C5—C4—C5'	86.2 (3)	C5'—C3A'—N3'—C17	−175.0 (2)
C3A—C5—C4—C5'	−92.6 (3)	C2—N3—C3A—O3A	−178.5 (2)
C6—C5—C4—C7	−134.7 (2)	C14—N3—C3A—O3A	1.2 (4)
C3A—C5—C4—C7	46.5 (3)	C2—N3—C3A—C5	2.5 (4)
C6'—C5'—C4—C5	75.3 (3)	C14—N3—C3A—C5	−177.9 (2)
C3A'—C5'—C4—C5	−102.8 (3)	C6—C5—C3A—O3A	−175.5 (2)
C6'—C5'—C4—C7	−63.1 (3)	C4—C5—C3A—O3A	3.4 (4)
C3A'—C5'—C4—C7	118.8 (2)	C6—C5—C3A—N3	3.5 (4)
C3A—C5—C6—N15	173.9 (2)	C4—C5—C3A—N3	−177.7 (2)
C4—C5—C6—N15	−4.9 (4)	C3A—N3—C2—O2	176.5 (3)
C3A—C5—C6—N1	−6.9 (4)	C14—N3—C2—O2	−3.2 (4)
C4—C5—C6—N1	174.3 (2)	C3A—N3—C2—N1	−4.8 (4)
C2—N1—C6—N15	−176.2 (2)	C14—N3—C2—N1	175.5 (2)
C13—N1—C6—N15	5.1 (3)	C6—N1—C2—O2	−180.0 (3)
C2—N1—C6—C5	4.6 (4)	C13—N1—C2—O2	−1.3 (4)
C13—N1—C6—C5	−174.1 (2)	C6—N1—C2—N3	1.3 (4)
C3A'—C5'—C6'—N18	176.0 (2)	C13—N1—C2—N3	−179.9 (2)
C4—C5'—C6'—N18	−2.1 (4)	C3A'—N3'—C2'—O2'	−177.2 (3)
C3A'—C5'—C6'—N1'	−6.9 (4)	C17—N3'—C2'—O2'	0.2 (4)
C4—C5'—C6'—N1'	175.0 (2)	C3A'—N3'—C2'—N1'	1.8 (4)
C2'—N1'—C6'—N18	−171.0 (2)	C17—N3'—C2'—N1'	179.3 (2)
C16—N1'—C6'—N18	6.6 (4)	C6'—N1'—C2'—O2'	170.1 (2)
C2'—N1'—C6'—C5'	11.7 (4)	C16—N1'—C2'—O2'	−7.6 (4)
C16—N1'—C6'—C5'	−170.7 (2)	C6'—N1'—C2'—N3'	−8.9 (4)
C5—C4—C7—C8	−142.5 (2)	C16—N1'—C2'—N3'	173.4 (2)
C5'—C4—C7—C8	−3.2 (3)	C8—C7—C12—C11	1.4 (4)
C5—C4—C7—C12	44.9 (3)	C4—C7—C12—C11	174.4 (2)
C5'—C4—C7—C12	−175.8 (2)	C12—C7—C8—C9	−2.7 (4)
C6'—C5'—C3A'—O3A'	179.7 (2)	C4—C7—C8—C9	−175.4 (2)
C4—C5'—C3A'—O3A'	−2.0 (4)	C9—C10—C11—C12	−1.6 (4)
C6'—C5'—C3A'—N3'	0.1 (4)	F20—C10—C11—C12	177.9 (2)
C4—C5'—C3A'—N3'	178.3 (2)	C7—C12—C11—C10	0.7 (4)
O3A'—C3A'—N3'—C2'	−177.2 (2)	C11—C10—C9—C8	0.3 (4)
C5'—C3A'—N3'—C2'	2.4 (4)	F20—C10—C9—C8	−179.2 (2)
O3A'—C3A'—N3'—C17	5.4 (3)	C7—C8—C9—C10	1.9 (4)

### Hydrogen-bond geometry (Å, º)

Cg is the centroid of the C7–C12 ring.

**Table:**

*D*—H···*A*	*D*—H	H···*A*	*D*···*A*	*D*—H···*A*
N15—H40···O3*A*′	0.96 (3)	1.96 (3)	2.916 (3)	174 (2)
N18—H50···O3*A*	0.93 (3)	1.88 (3)	2.803 (3)	170 (2)
N15—H30···O3*A*^i^	0.86 (3)	2.26 (3)	3.083 (3)	161 (3)
N18—H60···O3*A*′^ii^	0.91 (3)	2.14 (3)	3.007 (3)	159 (2)
C13—H13*A*···O3*A*^i^	0.96	2.41	3.154 (3)	134
C13—H13*A*···*Cg*^iii^	0.96	2.98	3.744 (3)	138

Symmetry codes: (i) *x*+1/2, −*y*+3/2, −*z*+1; (ii) −*x*+1/2, *y*−1/2, *z*; (iii) *x*−1/2, −*y*+3/2, −*z*+1.
