# Supplementary figures and images for: Crystal structure of 5,5′-[(4-fluoro­phen­yl)methyl­ene]bis­[6-amino-1,3-di­methyl­pyrimidine-2,4(1H,3H)-dione]
Source: Acta Crystallogr Sect E Struct Rep Online. 2014 Sep 10;70(Pt 10):o1098–9. doi: 10.1107/S1600536814019886 (PMC4257154; doi:10.1107/S1600536814019886)

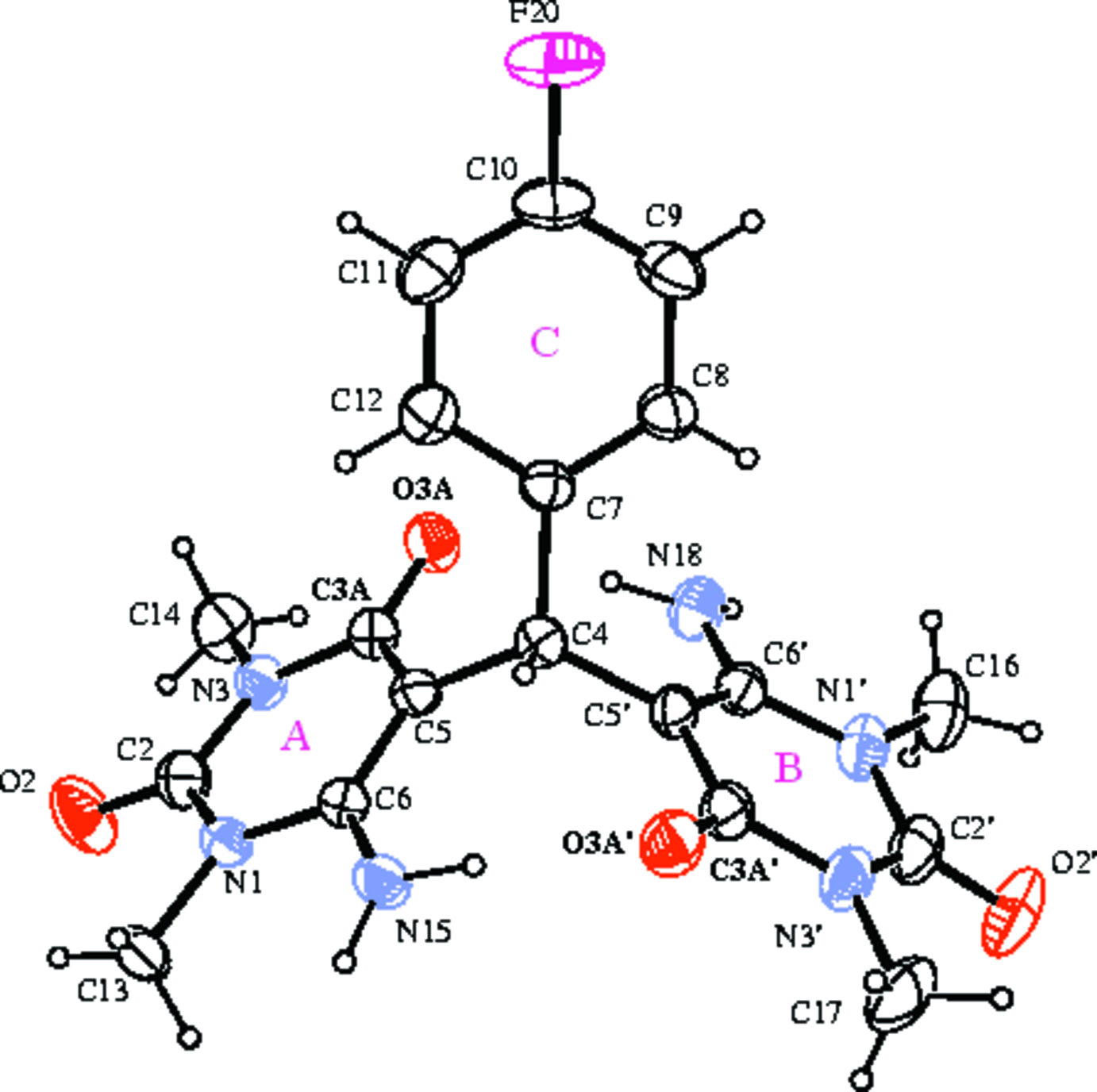

Supplement: Supplementary file 4 [file e-70-o1098-fig1.tif]

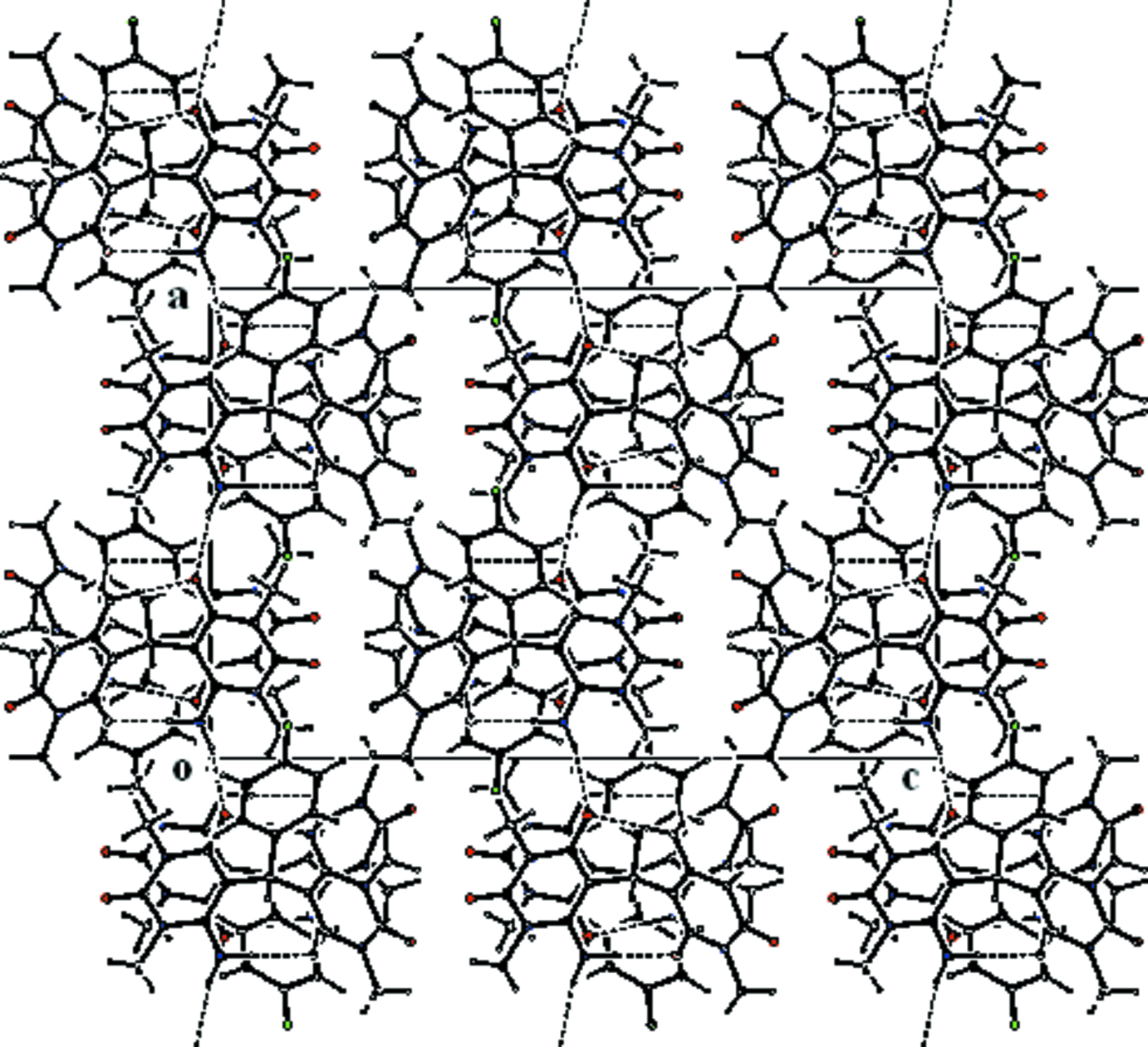

Supplement: Supplementary file 5 [file e-70-o1098-fig2.tif]
